# MS360°: a conceptual digital-first, data-driven hybrid care framework for personalised multiple sclerosis management

**DOI:** 10.1038/s41746-026-02461-4

**Published:** 2026-03-06

**Authors:** Isabel Voigt, Lars Masanneck, Marc Pawlitzki, Hernan Inojosa, Sven G. Meuth, Tjalf Ziemssen

**Affiliations:** 1 Center of Clinical Neuroscience, Department of Neurology, Faculty of Medicine and University Hospital Carl Gustav Carus, TUD Dresden University of Technology, Dresden, Germany; 2 Centre for Tactile Internet with Human-in-the-Loop (CeTI), TUD Dresden University of Technology, Dresden, Germany; 3 Department of Neurology, Medical Faculty, University Hospital Düsseldorf, Düsseldorf, Germany

**Keywords:** Engineering, Health care, Neurology

## Abstract

This perspective introduces MS360°, a conceptual hybrid care model for the management of multiple sclerosis (MS). It integrates traditional on-site assessments with digital health technologies (DHT) to enable more continuous, personalised, and proactive disease management. Current MS care is often fragmented, limiting timely interventions and patient engagement. MS360° addresses these challenges by introducing a digital-first hybrid framework for continuous data collection through remote monitoring, wearable sensors, and telemedicine. This data can be used to dynamically steer structured patient pathways and trigger targeted on-site assessments and interventions such as neurological examinations, imaging, laboratory assessments, and standardised functional tests based on predefined thresholds and patient profiles. The interaction of multidisciplinary teams, structured care pathways and bidirectional data flow enables timely clinical decision-making, stratified patient management and early detection of disease progression. Digital tools can further enhance patient engagement and lifestyle management, promoting adherence and outcomes. New technologies, including artificial intelligence and digital twins, are being discussed as potential future extensions for precision care, workflow optimisation, and risk prediction. MS360° provides a quality-driven conceptual framework, offering a roadmap for integrating digital innovations into patient-centred MS care.

## Introduction

Multiple sclerosis (MS) is a multifaceted disease that requires high-quality and personalised management, which is often hampered by existing healthcare structures^1,2^. Digital health technologies (DHTs), including electronic symptom assessments, telemedicine, digital biomarkers, and multidimensional tools for monitoring disease outcomes, have begun to reshape MS care^3^^,^^4^. These innovations show great potential for improving MS care and enhancing the efficiency and quality of disease management. Despite these promising prospects, integration into routine practice remains inconsistent, and many people with MS (pwMS) still do not receive personalised care^5^. A key limitation is that digital data are often collected without being systematically linked to clinical decision-making or the organisation of care pathways. Here, we present MS360°, a conceptual, digital-first hybrid MS care framework that integrates on-site and remote care components in a structured manner. MS360° is designed to enable continuous digital data acquisition as a foundation for more proactive, needs-based management of MS care, rather than limiting digital tools to episodic or passive use. By integrating structured care pathways, multidisciplinary teamwork, and digital tools, MS360° strives to meet both the complex needs of pwMS and the operational requirements of healthcare providers (HCPs). Key terms used throughout this manuscript are summarised in Supplementary Table 1.

## Current challenges in MS management

### Inefficiencies in care provision

Fragmented care structures and variable management practices can reduce the efficiency of MS care. Treatment by non-specialised neurologists may delay diagnosis and recognition of disease progression, resulting in postponed therapy initiation and an increased risk of more frequent and severe relapses^4^. In routine care, symptoms are often incompletely reported by pwMS and many hospital admissions could be managed more efficiently in outpatient settings^6,7^. Unnecessary on-site visits, duplicate examinations and uncoordinated follow-up strain healthcare resources and underscore the need for more efficient care models. These inefficiencies are exacerbated by episodic care approaches, in which infrequent on-site assessments may fail to detect relevant changes in symptoms or function between visits.

### Limited personalised and multidisciplinary care

MS progresses unpredictably and heterogeneously, requiring coordinated multidisciplinary care. However, many MS centres lack comprehensive care teams including neurologists, nurses and relevant specialists^8^. Consequently, care is often insufficiently tailored to individual needs, and pwMS experience long waiting times for assessments and treatment. The absence of structured longitudinal data further limits proactive, needs-based care planning.

### Restricted access to care and low patient engagement

Many pwMS are insufficiently engaged in their care and lack the resources to understand and manage their condition. This is particularly evident in rural areas or underserved regions, where access to specialised MS services and multidisciplinary teams is limited and travel to MS centres is burdensome^7,9,10^. Consequently, care often relies on sporadic face-to-face consultations, with limited structured support between visits, low health literacy, and few opportunities for shared decision-making.

### Lack of standardisation and structured monitoring

The absence of standardised care pathways and structured data collection leads to inefficient workflows and fragmented monitoring in MS care. Existing guidelines focus on individual aspects of management, while robust, patient-oriented quality indicators are largely lacking and recommendations from multiple advisory bodies have become increasingly complex^1,11^. In contrast to other medical disciplines, MS care does not follow a target-based, standardised monitoring framework^4^. Consequently, there is no consensus on what data should be collected, at what frequency, and with what level of detail. As a result, digital data collection in MS remains largely ad hoc, heterogeneous, and insufficiently integrated into routine care.

These challenges underscore the need for a more structured approach to MS management. Current care remains largely episodic, with digital tools applied inconsistently and monitoring practices unstandardised. A hybrid care model combining digital-first longitudinal data collection with clearly defined mechanisms to guide patient pathways and on-site care is therefore required to enable a more standardised, accessible, and patient-centred MS management.

## A conceptual framework for hybrid MS care – MS360°

Current MS care increasingly combines traditional clinical assessments with digital tools, yet these elements often remain disconnected in routine practice. MS360° bridges this gap by offering a structured framework that integrates clinical, organisational, and digital elements into a unified hybrid model. A core principle is the digital-first approach to acquiring longitudinal data, whereby continuously collected remote data informs the timing, intensity, and type of on-site assessments and interventions. Within this framework, DHTs are not standalone add-ons, but integral components that guide structured care pathways, trigger clinical reviews based on predefined thresholds, and coordinate care between HCPs and pwMS, while supporting engagement and self-management^12^. The following sections outline the key components of hybrid MS care. “Organisational foundations of hybrid MS care” to “Remote and digital care components” describe the organisational, clinical, and digital building blocks, while the following section integrates these elements into a data-driven hybrid care pathway that steers on-site care dynamically.

### Organisational foundations of hybrid MS care

Effective hybrid MS care relies on organisational elements that support coordinated, continuous, and high-quality management across settings. These include multidisciplinary teams – MS neurologists, nurses, neuropsychologists, clinical psychologists, physiotherapists, occupational therapists and administrative staff – working together with additional specialists such as speech therapists, spasticity experts or pain specialists^8^. Structured care pathways guide the sequencing of diagnostic and therapeutic steps, ensuring consistent documentation, monitoring, and evaluation of the care process^2^. In conjunction with digital monitoring and communication solutions, multidisciplinary teams and care pathways provide the basis for hybrid MS care. In MS360°, these organisational foundations are designed to support continuous digital data flows and clearly assigned responsibilities for reviewing, interpreting, and acting upon remotely generated signals.

### On-site clinical care components

On-site care encompasses all clinical procedures requiring the patients’ physical presence, such as anamnesis, neurological examination, and standardised functional assessments (e.g. Expanded Disability Status Scale [EDSS], Multiple Sclerosis Performance Test [MSPT]), as well as imaging and laboratory diagnostics (magnetic resonance imaging [MRI], blood, urine, cerebrospinal fluid)^3,13–15^. Advanced diagnostics may include optical coherence tomography (OCT) and visual evoked potentials (VEP) for optic nerve assessment, detailed gait analysis, and neuropsychological testing^16–18^. Not all assessments are performed at every visit; selection and frequency are tailored to patient needs, disease stage, and resources. In MS360°, on-site assessments are selectively deployed based on clinical need and signals emerging from longitudinal remote monitoring.

On-site evaluations enable treatment to be tailored to disease stage, disability and individual factors. MS management typically includes: acute relapse management (high-dose corticosteroids, plasmapheresis if necessary), disease-modifying therapies (DMTs, ranging from first-line injectables to highly effective agents), and symptomatic therapies that treat mobility impairment, fatigue, cognitive dysfunction, and other disease-related symptoms.

Many diagnostic, monitoring, and treatment procedures require specialised facilities (e.g., MRI scanners), sterile conditions (e.g., lumbar punctures, intravenous therapies) and immediate access to emergency care, highlighting the need for trained HCPs for safe administration and monitoring.

On-site visits provide access to comprehensive diagnostics, therapies, and interdisciplinary interaction. Their effectiveness depends on structured workflows, adequate staffing and specialised infrastructure. However, monitoring remains episodic, and access can be challenging for some patients^10^.

### Remote and digital care components

Remote care tools expand MS management by enabling frequent monitoring outside the clinic and complementing on-site visits^19^. Wearables and sensors continuously collect data to detect changes in depression symptoms, disability, and physiological parameters^20^. Validated smartphone applications support monitoring of motor, cognitive, and visual function, while digital platforms – such as that of *Icometrix*, combining a patient app (*icompanion*), an HCP portal and advanced MRI analysis – allow structured assessment of symptoms, disability, cognitive function. and fatigue^7^. Key characteristics, validation status, and recommended use frequencies of selected apps are summarised in Table 1, with evidence indicating reliable quantifications of functional aspects of MS^21,22^.

**Table 1:** Selected digital applications for MS monitoring: functionality, validation, data collection frequency, and EHR integration^21,22^

Application	Functional areas	Measurement methods	Validation status	Data collection frequency	EHR integration
**Floodlight**^70,71^	Dexterity, Mobility, Cognition	• Dexterity: finger pinching, shape drawing • Mobility: 2 min Walk Test, U-turn-test, static balance • Cognition: sSDMT	Moderate to strong, depending on domain (MS-specific validation studies available)	Daily or flexible	Possible
**MSCopilot** ^72,73^ (MS-specific digital monitoring app)	Walking ability, Dexterity, Cognition, Vision	• Walking: distance, speed • Dexterity: tracing different contours • Cognition: digit/symbol associations, information processing speed • Vision: low contrast visual function	Moderate to strong, depending on domain (MS-specific validation studies available)	Daily or flexible	Possible
**DIGICOG-MS**^74^	Cognitive domains: visuospatial and verbal memory, semantic fluency, information processing speed	• 4 digital tests: • remember and place • listen and repeat • generate words • associate numbers	Validation against standard tests: strong correlations between digital and traditional paper-based tests across all cognitive domains	Weekly/monthly	Possible
**iCompanion**^7,75–77^	Symptoms, Functions, Cognition, Fatigue (PROs)	• 12-item symptom battery • prEDSS for function • NeuroQoL Cognition and Fatigue	PROs validated (no MS-specific validation studies available for the entire app)	Flexible, regular PROs (weekly/monthly), relapses, and symptoms ad hoc	Possible

Validation status refers to available domain-specific MS studies; “Possible” in EHR integration indicates that data export can be achieved but may depend on local systems and interoperability.

*sSDMT* smartphone Symbol Digit Modalities Test, *prEDSS* patient-reported version of the Expanded Disability Status Scale, *NeuroQoL* Quality of Life in Neurological Disorders, *PRO* patient-reported outcome.

To ensure clinical relevance and safe integration into routine care, digital tools must meet established validation standards (analytical and clinical validity, clinical utility), and comply with regulations and data-protection requirements. These principles align with international frameworks for digital health interventions, such as the V3 for digital biomarkers and the mERA reporting standards^23–25^.

Telehealth has become an integral part of MS care. Video consultations enable routine follow-up appointments and urgent assessments, while asynchronous tools (e.g., secure messaging, results sharing, remote data transmission) support flexible monitoring between visits^6^. Remote care also reduces travel and logistical burdens. In Germany, digital support programs have improved access in rural settings, suggesting that integration with digital components could complement on-site care in MS^26^.

However, such digital tools often fail to translate into sustained patient engagement or meaningful changes in routine care without integration into structured care pathways and clearly defined clinical responsibilities. Limitations include variability in app validity, limited customisability, data security concerns, and the risk of patients discontinuing use. Effective remote care also depends on patients’ willingness to share data and sustainably integrate digital tools into their everyday lives. Industry-initiated apps may be withdrawn from the market^27,28^. In MS360°, remote and digital care components constitute the primary monitoring level, generating longitudinal data that feed into clinical review, prioritisation, and scheduling of on-site assessments, rather than remaining merely passive monitoring tools.

### Integration to MS360° - a digital-first, data-driven hybrid care model

MS360° integrates clinical, organisational, and digital elements into an implementation-ready, quality-driven hybrid care framework. Its key innovations of MS360° are summarised in Box 1. The following subsections describe its conceptual architecture and key components.

#### Concept and definition of MS360°

MS360° is a structured, digital-first, data-driven hybrid-care framework for pwMS that integrates standardised on-site assessments, continuous remote monitoring of mobility and PROs, rule-based alerts, multidisciplinary decision workflows, and integrated data and quality-management platforms. Rather than a single digital tool, MS360° represents a system-level model aimed to reduce unwarranted care variation, improve efficiency, and enable patient-centred disease management.

#### Context and rationale

Recent developments in neurological care illustrate how DHT, remote monitoring, biomarker data, and traditional diagnostics can be integrated into structured care pathways, particularly in cognitive disorders^29,30^. In MS, tele-visits, remote monitoring, telerehabilitation, and digital biomarker initiatives – many accelerated during and after the COVID-19 period – have improved access, continuity, and symptom tracking^31–35^. However, most initiatives remain domain-specific or research-focused and are not fully integrated in routine workflows. MS360° addresses this gap by systematically integrating digital tools into a multidisciplinary care pathway that combines remote and on-site assessments within a coherent, implementable hybrid care model^12,29^.

#### Operational model of MS360°

**Fig. 1:**
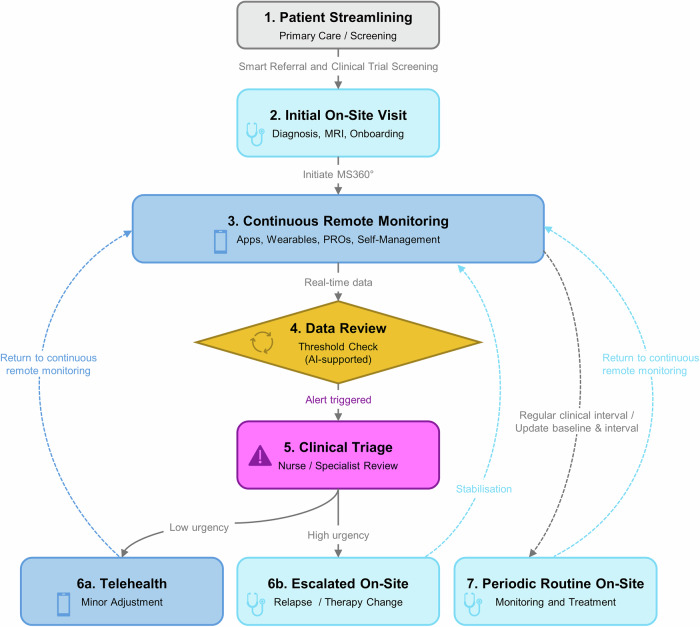
Hybrid MS Care Pathway. Patient identification and enrolment: Patients are identified and enrolled based on predefined clinical and organisational criteria. Initial on-site assessment: A comprehensive baseline evaluation is performed, including clinical, functional, cognitive, imaging, and laboratory assessments, and patients are introduced to the hybrid care pathway. Continuous remote monitoring: Longitudinal digital data, including patient-reported outcomes and sensor-based measures, are collected to support ongoing monitoring and self-management. Automated data review: Remote data are continuously or periodically analysed against predefined thresholds, supported by algorithm-based or AI-assisted analytics to prioritise signals and reduce data overload. Clinically relevant deviations trigger alerts when predefined criteria are met. Clinical triage and care coordination: Designated team members review alerts, coordinate care, and determine the appropriate response, including urgency and modality of follow-up. Hybrid intervention pathways: 6a. Telehealth management: Minor changes are addressed via teleconsultations and digitally supported therapies. 6b. Escalated on-site care: Clinically relevant deterioration prompts in-person reassessment or treatment adjustment. Scheduled routine on-site follow-up: Regular on-site visits are conducted independently of acute events to reassess disease status, update baseline data, and optimise long-term management. Following these visits, patients return to continuous remote monitoring. Across all steps, MS360° integrates structured coordination, planning, quality monitoring, and (AI-supported) data processing to ensure continuity, efficiency, and patient-centred care.

MS360° applies to patients with an established MS diagnosis after completion of standard diagnostic pathways before enrolment into hybrid care. The model delineates which assessments can be conducted remotely and which require specialised facilities (Table 2). Figure 1 illustrates a hybrid MS care pathway alternating between remote monitoring and on-site evaluations. Longitudinal remote data are reviewed at predefined intervals and thresholds, triggering alters, teleconsultations, or on-site visits as clinically indicated.

**Table 2:** Hybrid on-site and digital care components in MS360°

Component/Task	On-site care	Digital/Remote	MS360° integration
Diagnostics & Monitoring			
Basic MS Diagnosis	MS Specialist		Structured triage via hybrid pathway
Medical history - anamnesis	MS Specialist	e.g. with chatbot	PRO + remote intake
Neurological examination (EDSS)	MS Specialist	Remote follow-up possible	Threshold-based monitoring & alerts
Functional Testing (MSPT, gait)	With appropriate equipment	Continuous digital monitoring	Data-driven on-site scheduling
Physiological tracking	On-site assessment if needed	self-reported via apps	Continuous remote monitoring
PRO Collection (Fatigue, QoL, Cognition)	Initial on-site	Apps/Telehealth	Integrated digital collection
Cognitive/neuropsychological assessment	MS Specialist/Neuropsychologist	Digital tests possible	Hybrid follow-up & partial remote monitoring
MRI/Imaging (e.g., OCT, VEP)	Requires radiological infrastructure /specialized equipment	-	Hybrid workflow; remote interpretation optional
Lab Tests/Biomarkers	Laboratory facilities required	-	Structured reporting & alerts
Treatment			
DMT Initiation & Monitoring	MS Specialist (infusions and monitoring of side effects)	Remote follow-up - If stable	Hybrid decision-making & tiered intervention
High-efficacy DMT	MS Specialist (risk/benefit assessment)	-	Expert-led hybrid pathway
Adverse effects/comorbidities	MS Specialist (complex safety profiles)	Escalation protocols	Hybrid alert system & triage
Evaluation of treatment failure	MS Specialist (expert opinion on escalation)	-	Threshold-based alerts
Rehabilitation/Therapy			
Neuropsychological Training/Psychotherapy	For complex cases, standardised conditions required	Digital therapy/apps	Blended digital + on-site care
Physiotherapy/Occupational Therapy/Speech Therapy/Dietary Advice/Social Counselling	For initial assessment and complex cases	Remote exercises/consultations	Hybrid rehabilitation/lifestyle support
Patient-Cantered Care			
Education & Adherence	Initial on-site	Telehealth/apps	Continuous engagement & feedback
Self-Management Support	-	Digital tools	Personalised plans & monitoring
Coordination & Planning			
Multidisciplinary Coordination	Team meetings	Telemedicine possible	Defined roles & hybrid workflow
Clinical trials/Recruitment	On-site/specialised centres	Limited remote support	Hybrid integration & data coordination
Long-term care planning	MS Specialist	-	Continuous hybrid pathway

The table categorizes key examinations, assessments, and interventions in MS care, distinguishing between tasks performed on-site and those feasible digitally or remotely. Some assessments—such as basic MS diagnosis, MRI, lumbar puncture, and comprehensive cognitive testing—require specialised equipment or expert evaluation and are primarily conducted in-person. Other components, including PRO collection, symptom tracking, telehealth follow-ups, and hybrid interventions like neuropsychological training, physiotherapy, or lifestyle support, can be delivered remotely or in combination with on-site care. Notably, NeurostatusSMARTCARE enables the EDSS to be conducted via telemedicine by trained HCPs, expanding access and facilitating coordinated care and trial participation^78^.

The table illustrates a hybrid care model in which on-site assessments alternate with remote monitoring. Initial visits establish diagnosis and treatment plans, while patient status is continuously tracked using remote assessments, sensors, and questionnaires. Predefined thresholds trigger alerts for additional on-site evaluation if needed, ensuring timely, coordinated, and patient-centred care.

(*DMT* Disease Modifying Therapy, *EDSS* Expanded Disability Status Scale, *MSPT* Multiple Sclerosis Performance Test, *QoL* Quality of Life, *OCT* Optical Coherence Tomography, *PRO* Patient-Reported Outcome, *VEP* Visual Evoked Potentials).

#### Core features of MS360°

##### Integrated hybrid pathway and multidisciplinary team

Multidisciplinary teams coordinate care through structured pathways. Designated team members identify patients suitable for hybrid care based on disease stage, disability, digital literacy, internet access, and motivation. Transparent application of criteria, with ethical oversight, mitigates bias and promotes equitable inclusion, particularly for underserved or rural populations.

Clearly defined team structures and responsibilities form the organisational backbone of MS360°, ensuring coordinated decision-making across on-site and remote care settings.

##### Bidirectional data flow and decision-logic

Hybrid care relies on bidirectional flows: remote monitoring data trigger clinical reviews based on predefined thresholds such as clinically meaningful changes in gait speed (>20% deviation from baseline), reductions in cognitive performance exceeding test-specific minimal clinically important differences, or PRO scores surpassing validated cutoff values for fatigue, depression, or functional decline while on-site visits contextualise and refine digital signals^7,36,37^. This approach supports structured triage and ensures treatment decisions integrate continuous digital data with clinical assessment^25^.

##### Workflow integration and resource optimisation

While hybrid care can improve access and engagement, evidence, particularly from the US, indicates that telemonitoring may increase HCP workload due to additional data review, alerts, and administrative tasks^38–40^. Structured team roles, automated triage, and integrated protocols may mitigate these challenges. For example, MS nurses review remote data, prioritise alerts by clinical urgency, and embed documentation into existing clinical systems, reducing cognitive and administrative burden and ensuring telehealth complements on-site assessments. A conceptual tiered approach could help optimise workload: patients self-monitor via digital tools (tier 1), automated systems flag issues (tier 2), and clinicians intervene only for relevant alerts (tier 3).

Under these conditions, hybrid MS care can enhance efficiency by complementing on-site visits with telehealth for routine monitoring and minor consultations, reducing travel, optimising the frequency and intensity of on-site assessments, and preserving clinic capacity for urgent cases^19^. Stratified care prioritises high-need patients while supporting self-management. Evidence from MS telerehabilitation indicates that remote monitoring can optimise resource allocation without compromising clinical quality, provided that appropriate staffing models, workflow adaptation, and patient support are in place^2,41–44^. Digital tools may further streamline clinical workflows through automated documentation and data integration, enabling clinicians to make more data-driven decisions and earlier interventions^25^. Artificial intelligence (AI) may assist in identifying suitable candidates for clinical trials or targeted interventions^45^. Secure patient portals and dashboards facilitate patient engagement by providing access to health information, treatment recommendations and study opportunities^46^. Beyond efficiency gains, continuous remote monitoring enables pwMS to track their health trajectories, access relevant data, and participate more actively in care decisions, thereby supporting continuity and flexibility of care^7^.

##### Patient-centred care, engagement and self-management

Periodic on-site visits maintain personal interaction for shared-decision making and delivery of non-digital therapies. This combination of support enables patients to reap the benefits of technology while retaining the human touch. A comprehensive treatment approach requires the integration of lifestyle management, including physical activity, nutrition, and comorbidity care, through scalable digital interventions such as digital health applications and digital therapeutics. Digital offerings can enable personalised care for comorbidities, improve information flow, and provide individualised recommendations through feedback mechanisms that foster motivation and adherence^47^.

##### Quality management, data platforms, and standardisation

Digital hybrid pathways enable systematic quality management by capturing treatment processes, outcomes, and patient experiences in structured formats^1^. Integrated data platforms support the development and monitoring of quality indicators by providing HCPs with clear, actionable overviews of relevant data^5^.

Overall, hybrid care models such as MS360° combine digital innovation with the strengths of traditional healthcare, creating a flexible, efficient, and patient-centred ecosystem for MS care. Table 3 summarises key challenges and illustrates how MS360° addresses them.

**Table 3:** Key challenges in MS management and MS360° solutions

Challenge in current MS care	MS360° approach/solution
**Inefficiencies in care provision** (diagnostic delays, under-reporting, unnecessary hospitalisations, redundant visits)	Digital-first longitudinal monitoring feeds into structured on-site assessments and automated alerts. Integrated hybrid care pathways reduce unnecessary visits, optimize scheduling, and prioritise high-need patients.
**Limited personalised and multidisciplinary care**	Multidisciplinary team embedded in the hybrid model; individualised care plans informed by combined on-site and digital data; digital tools support coordination and timely interventions.
**Restricted access to care and low patient engagement**	Telehealth, mobile apps, and digital platforms increase access, enable patient self-management, and provide continuous engagement. Remote monitoring supports equitable access for rural or underserved populations.
**Lack of standardised care pathways and structured monitoring**	Standardised digital pathways integrate clinical workflows, dashboards, and quality indicators. Predefined thresholds and alerts guide clinical decisions, ensuring structured, consistent, and data-driven care.

By integrating a digital-first layer, structured hybrid pathways, and continuous remote monitoring with on-site assessments, MS360° addresses the main challenges in MS care. Remote data collection triggers alerts and guides scheduling of on-site visits, enabling timely interventions, patient-centred care, and efficient use of resources. Continuous engagement via telehealth, apps, and dashboards supports self-management, equity, and multidisciplinary coordination.

Box 1 Key innovations of MS360° MS360° consolidates hybrid MS care into an implementation-ready, quality-driven framework. Its novelty derives from five core elements:

1. **Integrated hybrid pathway** – Combines on-site assessments, remote monitoring, PRO collection, and telehealth follow-up into a structured pathway, ensuring coordinated, data-driven care.
2. **Threshold-based, bidirectional data flow –** Digital measurements (e.g., gait deviations, cognitive changes, PRO cutoffs) trigger timely clinical interventions, while on-site assessments refine interpretation.
3. **Workflow standardisation and resource optimisation –** Defined roles, automated documentation, and triage logic enhance efficiency and reduce unnecessary visits.
4. **Patient-centred digital support –** Individualised care plans integrate remote monitoring, education, and self-management tools to improve engagement and access.
5. **Continuous quality management –** MS-specific quality indicators, dashboards, and iterative review cycles enable routine evaluation and improvement of hybrid care.

## Implementation barriers and enablers for hybrid care

While the hybrid MS care model illustrates how digital and on-site elements can be integrated to improve patient monitoring, decision-making, and workflow efficiency, successful implementation depends on addressing practical, regulatory, and organisational challenges. The following chapter outlines key barriers and enablers for deploying such models in real-world clinical practice, including reimbursement structures, data protection, interoperability, workforce capacity, and patient engagement.

### Reimbursement and economic incentives

Implementing hybrid MS care requires not only technical feasibility but also appropriate reimbursement and economic incentives. Traditional fee schedules often prioritise on-site visits, while teleconsultations, remote monitoring, and digital therapies are variably reimbursed, which can discourage workflow redesign, adoption of new tools, and staff training, even when the clinical rationale is compelling. Conversely, structured reimbursement for telemedicine, remote monitoring, and approved digital health applications – particularly when combined with value-based payment models that reward outcomes rather than visit numbers – can incentivise implementation^48^.

Training and incentives for HCPs may be provided through governmental programs, professional societies, healthcare providers, or institutional initiatives, depending on the local healthcare context. Once clinical effectiveness is demonstrated, telemonitoring may become reimbursable, as seen in other chronic diseases such as heart failure. This, in turn, enables structured training and support for HCPs, which are critical for sustainable implementation but vary across healthcare systems and local contexts^49,50^. Assessing cost-effectiveness, including personnel, IT infrastructure, and training requirements, and clearly defining remuneration for specific activities are prerequisites for sustainable implementation and policy adoption.

### Data protection regulation and interoperability

Hybrid care depends on seamless data exchange between patients, devices, apps, electronic health records (EHR), and clinical information systems. Compliance with data privacy and security regulations, consent management, and support for interoperability standards (e.g. Fast Healthcare Interoperability Resources [FHIR], Systematised Nomenclature of Medicine – Clinical Terms [SNOMED CT]) are essential to integrate data from digital tools into EHR and clinical workflows^28^. A lack of interoperability between proprietary platforms can lead to data silos and duplicate documentation, undermining the efficiency gains promised by hybrid care. Clear regulatory frameworks, harmonised data protection standards, and incentives for open, interoperable interfaces are important prerequisites. Without them, hybrid care could increase rather than reduce workload for HCPs.

The rapid emergence of AI-powered tools and digital therapeutics arises additional ethical, legal, and regulatory questions. These include data governance, transparency of algorithms, explainability of AI-based decision support, and adherence to medical-device regulatory pathways such as the EU AI Act and the Medical Device Regulation (MDR), which define risk management, clinical evaluation, and reporting requirements for high-risk AI systems^51^. Current AI-based systems cannot reliably generate accurate, guideline-based answers for specific neurological questions and may provide erroneous, outdated or potentially harmful recommendations^52^. Robust validation against clinical standards, transparent performance reporting, and regulatory oversight are required before safe integration into hybrid care pathways. Ethical principles such as fairness, accountability, patient safety, and human oversight are essential to maintain trust and ensure responsible use. Liability and governance structures must also be clearly defined to support safe deployment and protect both patients and providers^53–56^.

### Workflow integration and change management

Beyond technology, hybrid care must fit into daily clinical practice. Hybrid models may initially increase healthcare utilisation due to more frequent remote data review. Staffing and workflow adaptions – such as designated telehealth coordinators or MS nurses reviewing incoming data – are therefore critical to prevent clinician overload. Clear role definitions, structured alert management, and patient onboarding protocols are essential.

Poor integration risks parallel processes, alert fatigue, cognitive load and unclear responsibilities. Training and ongoing support enable HCPs to select appropriate tools and respond to alerts while maintaining care quality. Concerns about increased workload, disrupted routines, and limited experience with DHT remain major barriers to adoption^28^. Demonstrating tangible benefits for patient outcomes and workflows, alongside financial incentives and protected implementation time, is crucial^47^.

From an implementation-science perspective, MS360° aligns with key elements of established frameworks such as RE-AIM and NASSS by addressing reach and equity, organisational complexity, sustainability and transparent reporting of digital components and workflows^57–59^. These frameworks support critical reflection on adoption, scalability, and long-term implementation.

### Digital literacy, patient engagement and equity

Patients face multiple barriers to engaging with hybrid care, including access to technology, broadband connectivity, comfort with digital tools across age groups, socioeconomic strata, and geographic regions. Although many pwMS use mobile apps, usability issues, limited relevance, perceived burden, or inadequate connectivity can reduce adoption and adherence^28^. Digital tools, therefore, need to be accessible, intuitive and adaptable to varying levels of disability and digital literacy^9,28^. Clinicians likewise require digital competence to interpret and act on continuously collected data.

Maintaining the patient–HCP relationship remains essential, as trust and human connection are central to effective care; digital tools should augment rather than replace them. When shifting interaction to digital channels, clear communication, empathy, and counselling are crucial^28^.

Hybrid care must explicitly address equity by supporting patients with limited digital access or literacy through inclusive design, alternative care pathways, and supportive policies. By actively addressing these barriers, hybrid care can enhance inclusion and equity rather than exacerbating existing disparities^60^.

With appropriate infrastructure, interoperability, training, and change management strategies, the benefits of hybrid management – improved outcomes, efficiency, and satisfaction – can be realised without compromising the quality and human aspects of clinical practice.

## Implementation roadmap for MS360°

**Fig. 2:**
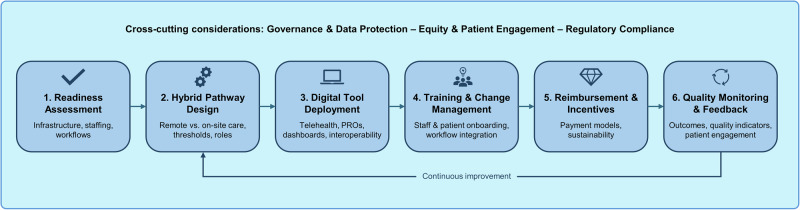
Implementation roadmap for MS360°. The figure illustrates a stepwise approach for implementing the MS360° hybrid care framework. (1) Starting with an assessment of organisational and technical readiness (infrastructure [digital, HER, connectivity], staffing and competencies, workflow mapping), (2) centres define structured hybrid care pathways (definition: remote vs. on-site assessments, escalation and alert thresholds, roles and responsibilities), (3) deploy interoperable digital tools (telehealth platforms, PRO tools, dashboards, interoperability and data governance, security and regulatory compliance), and (4) implement training and change management strategies (staff and patient training, workflow integration, communication and onboarding protocols). Alignment of (5) reimbursement models (fee schedules/value-based payment models, sustainability and cost-effectiveness considerations) and (6) continuous quality monitoring (collection and analysis of outcomes, patient engagement and quality indicators) support sustainability. The iterative feedback loop highlights continuous optimisation of hybrid MS care.

Implementing MS360° follows a structured, stepwise approach, as summarised in Fig. 2. Detailed elements, including organisational, technical, and patient-centred considerations, are illustrated in the figure legend.

### Future perspectives and conclusion

The near future will see more advanced DHTs in MS care, using AI and machine learning to analyse data to predict disease progression and inform treatment decisions^61^. These tools will improve and become faster, more detailed, and ate, enabling personalised medicine. Digital care pathways guide users through diagnosis, monitoring, and therapy, automatically documenting care activities and outcomes. These intelligent pathways could optimise clinical workflows and evaluate care quality and cost-effectiveness^2^.

A key emerging innovation is the digital twin (DT), a virtual representation of an individual patient, continuously updated with real-world clinical and lifestyle data^62^. Comparable frameworks have already been implemented in medical fields^63–66^. DT simulate disease trajectories, treatment responses, and risk profiles, supporting precision medicine, trial recruitment, and therapy optimisation. However, clinical integration requires robust validation against clinical standards, adherence to regulatory frameworks (e.g. EU AI Act, MDR) and seamless incorporation into care workflows^67,68^. Known limitations, including potential algorithmic bias, incomplete data, and uncertainty in predictive accuracy, must be addressed to ensure patient safety, clinical reliability, and ethical deployment^53,54,69^.

Successful adoption of AI, DTs, and hybrid care models depends on supporting infrastructure, interoperable systems, data governance, and workflow adaptation. When these prerequisites are met, hybrid management can enable more proactive, personalised, and responsive MS care, with potential improvements in outcomes and quality of life for pwMS.

## Supplementary information

Supplementary Information

## Data Availability

No datasets were generated or analysed during the current study.
