# Supplementary material for: MS360°: a conceptual digital-first, data-driven hybrid care framework for personalised multiple sclerosis management
Source: NPJ Digit Med. 2026 Mar 6;9:229. doi: 10.1038/s41746-026-02461-4 (PMC12996551; doi:10.1038/s41746-026-02461-4)
Supplement: Supplementary file 1 — Supplementary Information [file 41746_2026_2461_MOESM1_ESM.docx]

Supplementary Table: Glossary of Terms

| **Term** | **Explanation** |
| --- | --- |
| Artificial intelligence (AI) in healthcare | complex algorithms and software that mimic human cognition in analyzing, interpreting and understanding complex medical data |
| Bidirectional Feedback | two-way communication mechanism in hybrid care models allowing both patients and healthcare providers to exchange data and insights continuously, enhancing shared decision-making and therapy adjustment. |
| Dashboard | visual tool for displaying key performance indicators and supporting data-based decisions in the healthcare sector; components |
| DiGA | digital therapeutic applications in the German healthcare system that can be prescribed by doctors and reimbursed by health insurance companies |
| Digital Biomarkers | objective, quantifiable physiological and behavioral data collected through digital devices that can be used to monitor health status and disease progression in real time. |
| Digital Health Technologies (DHT) | comprehensive digital platforms, connectivity, software and sensors to improve healthcare. This includes mHealth, health IT, wearables, telehealth and personalized medicine |
| Digital Patient Pathway | a structured digital framework that guides patients and providers through diagnosis, treatment, and follow-up processes using automated data collection, alerts, and decision-support tools. |
| Digital Phenotyping | the moment-by-moment quantification of individual-level human behavior in health and disease, using data collected from personal digital devices |
| Digital Platform | basic technological infrastructure for the integration of various digital health technologies |
| Digital Therapeutics (DTx) | evidence-based digital products for the treatment of diseases, disorders or injuries with demonstrable therapeutic effects |
| Digital Twin (DT) | virtual representation of physical systems such as organs, physiological systems or entire patients that dynamically integrate real-time data to map individual characteristics |
| Electronic Health Records (EHR) | digital patient records for storing and managing health information |
| Health Literacy | the ability of individuals to access, understand, and use information to make informed health decisions; a critical factor in the successful adoption of digital health tools |
| Human-Centered Design | design approach that prioritizes the needs, preferences, and limitations of the end-users—especially patients and HCPs—in the development of digital health applications |
| Hybrid care model | combined care model comprising digital and personal healthcare services |
| Integrated Care | care that coordinates various healthcare services |
| Interoperability | ability of different systems and technologies to work together and exchange data |
| Machine Learning (ML) in healthcare | sub-area of AI that includes algorithms that automatically improve through experience, categorized into supervised learning, unsupervised learning and reinforcement learning |
| Mobile Health (mHealth) | use of mobile devices such as smartphones and tablets to support medical and public health practices, including health monitoring, education, and communication |
| Patient empowerment | strengthening patient autonomy and participation |
| Patient Portal | online platform for patients to access their health data and communicate with HCPs |
| Patient-Centered Care | care that is geared towards individual needs of patients |
| Patient-Reported Outcome (PRO) | health outcomes reported by patients themselves |
| Quality Indicators in MS Care | measurable elements of clinical practice performance used to assess the quality and outcomes of multiple sclerosis care |
| Real world setting | clinical environment outside of controlled study conditions in which regular healthcare takes place, characterized by variable factors such as diverse patient populations, different treatment regimens and external influences |
| Remote monitoring | observing patient data and health parameters outside of clinical facilities |
| Self-management tools | tools for independent health monitoring |
| Telemedicine | remote treatment and consultation of patients by healthcare professionals using telecommunications technology |
| Wearables | devices worn on the body for continuous monitoring of various health parameters |

This glossary contains key terms and definitions from the field of hybrid healthcare and digital health technologies
